# Editorial: Advancing high-resolution 3T MRI for cognitive and clinical neuroscience

**DOI:** 10.3389/fnins.2026.1822879

**Published:** 2026-03-26

**Authors:** Sriranga Kashyap, Atena Akbari, Irati Markuerkiaga, David G. Norris, Kâmil Uludag, Maria Guidi

**Affiliations:** 1 Physical Sciences Platform, Sunnybrook Research Institute, Toronto, ON, Canada; 2 Krembil Brain Institute, University Health Network, Toronto, ON, Canada; 3 Centre for Functional and Metabolic Mapping, Robarts Research Institute, University of Western Ontario, London, ON, Canada; 4 Signal Theory and Communications, Department of Electronics and Computing, Mondragón Unibertsitatea, Arrasate-Mondragón, Spain; 5 Donders Centre for Cognitive Neuroimaging, Radboud University, Nijmegen, Netherlands; 6 Harquail Centre for Neuromodulation, Hurvitz Brain Sciences Program, Sunnybrook Research Institute, Toronto, ON, Canada; 7 Department of Medical Biophysics, University of Toronto, Toronto, ON, Canada; 8 Enrico Fermi Research Center, Rome, Italy

**Keywords:** 3 T fMRI, arterial spin label (ASL) MRI, brainstem, denoising, echo volumar imaging, gSLIDER, high resolution fMRI, VASO

Some of the most consequential technical developments in MRI over the past decade, including mesoscale imaging of cortical layers, columns, and fine-grained brain structures, have been predominantly explored at ultra-high field strengths (UHF, ≥7 T), where signal-to-noise (SNR) ratio advantages and enhanced signal sensitivity support very high spatio-temporal resolutions. During the same period, 3 T research increasingly prioritized standardized acquisition protocols, particularly in large-scale consortia and clinical research, emphasizing improved robustness, reproducibility, and cross-site comparability, at the cost of reduced incentives for methodological work aimed at pushing spatial and temporal boundaries. Consequently, certain high-resolution and high-specificity techniques have been less extensively developed or adopted at 3 T, reinforcing an implicit division between UHF systems used for methodological innovation and 3 T systems reserved for routine imaging. This divide is now being actively challenged, as a growing body of work demonstrates that advances in hardware, acquisition design, reconstruction, denoising, quantification, and registration can substantially extend the capabilities of 3 T MRI, enabling investigations previously considered to require UHF scanners whilst benefiting from the global ubiquity and improved patient comfort of 3T.

This Research Topic collects six contributions from 44 authors that highlight how novel acquisition strategies and pre-processing approaches can be leveraged to improve sensitivity, specificity, and interpretability in 3 T imaging, collectively advancing high spatio-temporal MRI at 3 T as a practical standard for cognitive and clinical neuroscience.

Two contributions address the acquisition and reconstruction axis directly, targeting the spatial and temporal resolution limits of 3 T functional MRI. Townsend et al. present gSLIDER-SWAT, pairing gSLIDER with a sliding-window reconstruction strategy to achieve up to 5-fold improvement in effective temporal resolution relative to the native gSLIDER repetition time. They validate this approach using a visual hemifield paradigm and apply it to naturalistic stimuli designed to elicit joy, reporting activation across limbic, striatal, and prefrontal regions, including basolateral amygdala subnuclei, that are often difficult to resolve at 3 T with conventional gradient-echo EPI. The paper offers a concrete acquisition-plus-reconstruction pathway for high-resolution, whole-brain fMRI at 3 T, while noting slab-boundary artifacts and temporal autocorrelation as open practical challenges. Posse et al. pursue a complementary goal with multi-band echo-volumar imaging (MB-EVI), a high-speed 3D acquisition framework that supports sub-second temporal resolution at millimeter-scale spatial resolution with real-time reconstruction capability. They show that MB-EVI enables sensitive mapping of both task-evoked activity and resting-state connectivity at short TRs, captures connectivity across both low- and higher-frequency bands, including above 0.3 Hz, and benefits from NORDIC denoising through substantial gains in temporal SNR without added blurring. The real-time compatibility of MB-EVI positions it as relevant not only for cognitive neuroscience but also for neurofeedback and clinical monitoring.

Improving spatial and temporal resolution, however, is only part of the challenge. The interpretability of functional maps depends critically on the contrast mechanism employed and on how noise is managed in the resulting time series. Zamboni et al. demonstrate this by deploying optimized cerebral blood volume (CBV)-sensitive VASO at 3 T to probe mesoscopic functional organization in human visual cortex. They report evidence for curvature-selective domains in area V4 consistent with prior findings in non-human primates, and argue that VASO yields more clearly defined modular organization than conventional BOLD, whose venous weighting can reduce spatial specificity and obscure fine-grained functional architecture. The result is both a neuroscience advance, evidence of curvature domains in V4, and a methods demonstration that layer-resolved, non-BOLD fMRI is feasible at 3 T. As the authors note, however, VASO carries inherently lower detection sensitivity than BOLD, making effective denoising essential. This motivates the study by Knudsen et al., who systematically evaluate how NORDIC-PCA denoising performs on VASO time series at 3 T across a parameter space specific to VASO. Their central conclusion is conditional but practically important: with appropriate implementation choices, NORDIC reduces thermal noise with minimal bias and preserves spatial resolution, but performance varies materially with parameter settings, and maximal noise removal does not necessarily correspond to best signal preservation. This paper functions as a methods validation and technical guide that translates denoising from a plug-and-play method into an empirically constrained one, whose consequences differ depending on whether the scientific goal is localization, anatomical reference, or quantitative inference across conditions.

The final two contributions address a different limitation altogether: the reliability of the parameters and regions of interest extracted from high-resolution 3 T data. Kirk et al. introduce Structured Stochastic Variational Bayes (SSVB), a gradient-based variational inference approach for quantifying cerebral blood flow and arterial transit time (ATT) from arterial spin labeling data. In simulations and high-resolution data from the Human Connectome Project Lifespan dataset, SSVB yields lower bias and improved robustness relative to existing approaches, with particular advantages for ATT estimation in low-SNR regimes such as white matter. By treating spatial smoothing as part of the optimisation rather than a fixed pre-processing decision, SSVB exemplifies how analysis-layer advances can complement acquisition-side innovations, improving interpretability of physiological parameters without erasing spatial structure. Chen et al. target a distinct but equally consequential source of error in brainstem studies: atlas-to-native ROI registration that relies on T1-weighted contrast poorly suited to resolving small, densely packed nuclei. They demonstrate that diffusion-derived images supply internal microstructural landmarks that improve brainstem alignment, and they introduce evaluation metrics applicable even when manual ground-truth delineations are not feasible. The contribution underscores that even optimal signal acquisition cannot rescue analyses when regions of interest are systematically mislocalised, a problem that multi-modal registration strategies can address.

## Outlook

Taken together, these contributions demonstrate that progress in 3 T neuroimaging now depends less on any single innovation and more on coordinated advances across the full methodological stack: acquisition and reconstruction to expand attainable spatiotemporal regimes, contrast choices that improve biological specificity, denoising strategies validated against the particular signal model in use, and inference and registration methods that stabilize estimation and localization in challenging anatomical territories.

We believe that 3 T MRI can plausibly become the practical standard for high spatio-temporal resolution neuroimaging, including in subcortex and brainstem, if methodological advances are paired with explicit, modality-specific validity checks that preserve biological interpretability while expanding what can be measured. The contributions assembled here demonstrate that this goal is not aspirational, but already underway, and we hope this Research Topic serves both as a resource for researchers seeking to push the boundaries of their own 3 T scanners and as a call for shared benchmarks and multi-site validation efforts that will be required to consolidate these advances into robust, reproducible practice.

